# COVID-19 in Ecuador: Imported Control Strategies without Context in a Challenged Healthcare System

**DOI:** 10.4269/ajtmh.20-1347

**Published:** 2020-11-17

**Authors:** Diego Herrera, Carlos Troya Altamirano, David Gaus

**Affiliations:** Andean Health & Development, Santo Domingo, Ecuador

A 54-year-old farmer with fever, shortness of breath, and body aches for the previous 5 days was brought into a triage tent at our rural hospital in Ecuador by his wife and 20-year-old daughter in early June of 2020. He was hypoxic, tachycardic, and clearly dyspneic. We had no access to RT-PCR COVID-19 testing. We were not alone.

The recent COVID-19 outbreak in our coastal city of Guayaquil, Ecuador, with its nearly three million inhabitants exposed many of the unique challenges low- and middle-income countries (LMICs) face. The number of COVID-19 cases began to climb drastically in March in Guayaquil; our government health system simply was not prepared. Hospitals saturated quickly, and wards were not designed for mass isolation. They suffered severe shortages of personal protective equipment (PPE) for healthcare personnel. One news source described that, despite early pandemic alerts, the central government purchased no PPE between January and March. Starting in January, medical supplies providers asked health officials to begin contractual processes to order PPE and testing materials, as this process requires many weeks. In April, no stock remained in central supply. On March 1, the minister of public health assured that hospitals had the medications and supplies to confront the pandemic. This excessive and false confidence led to the decision to allow a national soccer game to be played in Guayaquil with more than 30,000 in attendance on March 4. Many believe this was a super-spreader event.

In early June of 2020, when this family came to our nongovernmental hospital, not far from Guayaquil, we still had very little PPE and no testing capabilities. Few facilities in the country actually had regular access to testing. The patient rejected both the presumptive diagnosis of COVID-19 and hospital admission. On further interrogation of his wife and daughter, they both had cough and fever but were trying to hide their mild symptoms from us. Chest imaging confirmed the presumptive diagnosis of COVID-19 in all three of them. Why were they doing this?

After another hour of discussion and breaking down some barriers, we were able to ascertain some critical information. They all lived in a one-room home in a small community. They were unconvinced their malady was actually COVID-19 because without a confirmatory test, how could we be so certain of the diagnosis? They feared being admitted to a hospital because they were convinced there is a greater concentration of COVID-19 in hospitals and that they would surely die in the hospital, and were convinced that healthcare workers would make them sicker than they already were because of the paucity of personal protection equipment used by healthcare professionals. They were convinced that not only would they die in the hospital but also that no family members could be present with them. As they spoke, they frequently referred to the city of Guayaquil, leaving us with the distinct impression that what happened in Guayaquil impacted them profoundly.

In Guayaquil, 40% of healthcare workers were infected early in the outbreak. As of June 14, the Ecuadorian Medical Federation estimated that nationally, more than 120 physicians had died attending to COVID-19 patients, and that the number continues to rise. The government inappropriately declared that all healthcare personnel in hospitals would be provided hydroxychloroquine. As of September, PPE continues to be in very short supply. Knowing all of this, the family worried that even in our hospital providers would be in short supply.

Despite our reassurances to the contrary, the family also worried about the care they would receive and what equipment would be available. A recent report on ventilator purchases in Latin American countries placed Ecuador at the bottom of the list, with 57 purchased during the critical period. Paraguay, with less than half the population of Ecuador, followed with 63 purchased ventilators. However, seemingly limited experience with intensive care units (ICUs) in Guayaquil led to the mistaken notion that only mechanical ventilators were needed. In fact, the complexity of ICUs requires trained professionals, piped-in oxygen and compressed air, cardiorespiratory monitors, and medications—most of which remained unavailable in Guayaquil.

Patients with moderate/severe symptoms of COVID-19 quickly realized that going to hospitals was not worthwhile as beds and therapies were not available. The few working ventilators were unavailable to most patients who required them, so these patients returned home to die in the presence of their families.

Multiple family members living in impoverished settings, such as adjacent one- or two-room homes, made quarantine and social distancing an entirely unreachable prospect for most. Masks were essentially unavailable to the poor in Guayaquil, and conventional wisdom at that time suggested that only surgical masks could protect, not the homemade cloth masks. Handwashing was also a recommendation, with little hope for adherence from *Guayaquileños* living in shanty towns where piped water was rare.

Even after our government introduced control measures like social distancing, transportation presented obstacles. Most *Guayaquileños* use public transportation within the city. Forced to maintain safe distances in bus lines, people see those distances melt away as they board the bus. Domestic workers use these crowded buses daily to work in people’s homes, making them potentially significant disease spreaders. These conditions of squalor and overcrowding likely contributed to super-spreading of the infection.

The family also believed that if they died in the hospital, they would not be given a proper burial despite the availability of funeral services in our community. However, for several reasons, funeral home services were largely unavailable in the past months in Guayaquil. Undertakers feared contracting COVID-19 from cadavers. Severely limited cremation services thwarted recommendations that corpses be cremated. Limited funeral capability, the unavailability of hospital beds, and the outright fear of going to hospitals led to horrific scenes of cadavers being deposited on the streets and isolated folks dying alone in their homes, only to be found days later.

Limited testing worsened the problem. This, coupled with accumulation of corpses, rendered it impossible to ascertain an accurate number of deaths attributable to COVID-19 during the Guayaquil crisis. At one point, the government was reporting fewer than 200 officially confirmed total COVID-19 deaths, whereas the police were removing from the city more than 100 cadavers daily. At the peak of the Guayaquil outbreak, more than 400 deaths occurred per day.

PCR testing, although limited, was indeed available. Some private laboratories offered somewhat reasonable prices by U.S. standards (US$ 120), but minimum wage is approximately $475/month. Others gouged, up to US$ 500 per PCR test. With time, other testing became available, including antibody testing. Out of desperation, haste, and severely limited financing, antibody tests of unproven accuracy entered the market. Ecuador reached a mere 12 PCR tests per 1,000 inhabitants, the lowest rate in the region.

The shortage of testing and PPE also resulted from hoarding and overpricing. The media reported that long-standing corrupt practices of the health sector became even more acute as prices skyrocketed and hoarding increased.

We tried to persuade the family to stay. The father required 4 L of oxygen per minute to maintain his saturation, and we had available ICU beds with appropriately trained nursing staff available. But in the end, the family went home. We notified the ministry of public health officials responsible for contact tracing. We had no follow-up with the patient. Primary care physicians had little impact on the education of the community or the spread of the virus. As the community feared going to healthcare facilities, healthcare personnel did not conduct extension workout into the community. Physicians reported that contact tracing was not taught appropriately and therefore not implemented correctly by healthcare personnel. Primary healthcare strategy appeared to have less impact on behavior than social media.

The media carried stories about mathematical modeling that were mistakenly purported to accurately calculate the number of deaths that would result. This either worsened the already panicked population or led them to a false sense of security that conditions would normalize soon. Regular reports in the media described dubious therapies being used successfully. All the while, medical societies in Ecuador remained silent.

Following international recommendations, the government imposed curfews, lockdowns, and the closing of businesses. Those working in the informal market of Guayaquil, such as street vendors, transport workers, and domestic workers, make up a significant portion of the population and receive no unemployment benefits. Hit hardest by the lockdown in terms of jobs lost, they were least equipped to endure a work stoppage.

International media eventually learned of the crisis. The *New York Times* reported on April 23, 2020 that deaths were 15 times greater than those reported by Ecuador’s government. They calculated excess deaths from March 1 to April 15, 2020 to be 7,600, compared with 503 officially reported by the government.

Experts gathered data on mortality from Ecuador’s National Institute of Statistics and Census (INEC) and the Office of Civil Registration and updated it with surprising expediency. The city of Guayaquil and its province, Guayas, showed alarming death statistics during the critical period. The total COVID-19–related deaths in Guayaquil and the Guayas Province are best calculated through excess deaths over the normal average, with limitations. This number is now reported to be 11,729 and 14,438, respectively, and perhaps greater because of unidentified cadavers. But dengue, also endemic at this time in Guayas, may have contributed to excess deaths.

The rest of the country braced for the spread to occur, and it did. Ultimately, the *Financial Times* reported on September 2, 2020 that Ecuador was the hardest hit country in the world, with more than 1,000 excess deaths per million inhabitants.

The lack of testing has kept Ecuador off the global radar screen. Meanwhile, for many weeks, our neighbors to the north and south, Colombia and Peru, both ranked among the top five countries in the world for highest daily incidence of COVID-19 cases.

Our government did not report the extent of the crisis since its inception. Was this out of ignorance or by design? Either is unacceptable. The abysmal rates of PCR testing would suggest it was indeed by design. Hoarding and overpricing of test kits dramatically worsened the situation. Reports of rampant corruption within the healthcare system continued almost daily in the media.

In 2018, the Lancet Global Health Commission on High-Quality Health Systems in the Sustainable Development Goals Era discussed in its report the challenges healthcare systems in LMICs face to achieve quality services. The report did not discuss the impact of corruption. We authors have a combined 50-year experience in managing the operations of healthcare facilities in Ecuador, interacting daily with the public health system. We see the negative impact of the lack of accountability and transparency, leading to corruption, that permeates practically every level of healthcare delivery. In our experience, these issues weigh more heavily on negative outcomes than on any technical deficiencies of our healthcare system.

Conversely, the commission’s report described very accurately the need for greater leadership and the commitment of healthcare officials to return the focus of care to the patient, who seems to be lost in the public healthcare system. The COVID-19 crisis in Ecuador illustrates well that absence of leadership. It also calls into question many of the recommended protocols and guidelines put forth by agencies from high-income countries. How can a vision be created? How can leadership and responsibility be fostered? Why have Ecuadorian professional societies (medical, nursing, pharmaceutical, and others) remained silent throughout this crisis?

With the events of Guayaquil in the public eye, there remained little doubt as to why our patient and his family believed what they did. We continue to see patients who view COVID-19 through the lens of the events in Guayaquil. We also understand the limitations of our national healthcare system as it faces this pandemic, but those limitations have significant consequences.

